# Hypoxia-Induced Mitochondrial ROS and Function in Pulmonary Arterial Endothelial Cells

**DOI:** 10.3390/cells13211807

**Published:** 2024-11-01

**Authors:** Harrison Wang, Teng-Yao Song, Jorge Reyes-García, Yong-Xiao Wang

**Affiliations:** 1 Department of Molecular & Cellular Physiology, Albany Medical College, Albany, NY 12208, USAtengyaosong@gmail.com (T.-Y.S.); jreyes@facmed.unam.mx (J.R.-G.); 2 Departamento de Farmacología, Facultad de Medicina, Universidad Nacional Autónoma de México, Ciudad de Mexico 04510, Mexico

**Keywords:** hypoxia, pulmonary hypertension, pulmonary artery endothelial cells, nicotine

## Abstract

Pulmonary artery endothelial cells (PAECs) are a major contributor to hypoxic pulmonary hypertension (PH) due to the possible roles of reactive oxygen species (ROS). However, the molecular mechanisms and functional roles of ROS in PAECs are not well established. In this study, we first used Amplex UltraRed reagent to assess hydrogen peroxide (H_2_O_2_) generation. The result indicated that hypoxic exposure resulted in a significant increase in Amplex UltraRed-derived fluorescence (i.e., H_2_O_2_ production) in human PAECs. To complement this result, we employed lucigenin as a probe to detect superoxide (O_2_^−^) production. Our assays showed that hypoxia largely increased O_2_^−^ production. Hypoxia also enhanced H_2_O_2_ production in the mitochondria from PAECs. Using the genetically encoded H_2_O_2_ sensor HyPer, we further revealed the hypoxic ROS production in PAECs, which was fully blocked by the mitochondrial inhibitor rotenone or myxothiazol. Interestingly, hypoxia caused an increase in the migration of PAECs, determined by scratch wound assay. In contrast, nicotine, a major cigarette or e-cigarette component, had no effect. Moreover, hypoxia and nicotine co-exposure further increased migration. Transfection of lentiviral shRNAs specific for the mitochondrial Rieske iron–sulfur protein (RISP), which knocked down its expression and associated ROS generation, inhibited the hypoxic migration of PAECs. Hypoxia largely increased the proliferation of PAECs, determined using Ki67 staining and direct cell number accounting. Similarly, nicotine caused a large increase in proliferation. Moreover, hypoxia/nicotine co-exposure elicited a further increase in cell proliferation. RISP knockdown inhibited the proliferation of PAECs following hypoxia, nicotine exposure, and hypoxia/nicotine co-exposure. Taken together, our data demonstrate that hypoxia increases RISP-mediated mitochondrial ROS production, migration, and proliferation in human PAECs; nicotine has no effect on migration, increases proliferation, and promotes hypoxic proliferation; the effects of nicotine are largely mediated by RISP-dependent mitochondrial ROS signaling. Conceivably, PAECs may contribute to PH via the RISP-mediated mitochondrial ROS.

## 1. Introduction

Hypoxia-induced pulmonary hypertension (PH) is a severe and progressive lung disease characterized by increased pulmonary arterial pressure, eventually causing right ventricular failure and death [1,2,3,4,5]. Pulmonary artery insufficiency in PH can be triggered by various stimuli, such as hypoxia, inflammation, and vasoconstriction, leading to the proliferation and remodeling of pulmonary artery endothelial cells (PAECs) and pulmonary artery smooth muscle cells (PASMCs). PAECs are a critical component of the pulmonary vasculature and play an important role in maintaining vascular homeostasis [6]. Noticeably, profound changes in the function and phenotype of PAECs may occur, contributing to pulmonary vascular constriction, angiogenesis, inflammation, remodeling, and PH. These responses are highly associated with reactive oxygen species (ROS), which underlie the development and progression of the disease [7,8].

In general, two major endogenous sources of ROS are known, the family of NADPH oxidases (NOX) and electron chain transport (ETC) in the mitochondria [9]. The NOX genes give rise to transmembrane proteins that are required for electron transport across cell membranes. Upon activation, NOX catalyzes the transfer of electrons from NADPH to molecular oxygen, resulting in the formation of superoxide anions (O_2_^−^) [10,11]. In the mitochondria, ROS are formed as a by-product of oxidative phosphorylation, primarily through the loss of electrons from the ETC [12]. During oxidative phosphorylation, which occurs in the inner mitochondrial membrane, electrons are transferred along the ETC to eventually reduce O_2_ to H_2_O [2]. However, an inherent inefficiency of the ETC causes a small percentage of electrons to escape prematurely from the electron transport chain, promoting the formation of O_2_^−^ [13]. Superoxide anions are highly reactive and can generate other ROS, such as hydrogen peroxide (H_2_O_2_), by further dismutation [14]. Both enzymatically and non-enzymatically produced ROS are generated along the ETC [15]. Mitochondrial complexes I (NADH: ubiquinone oxidoreductase), II (succinate dehydrogenase), and III (cytochrome bc1 complex) all play roles in this enzymatic pathway. The enzymes NADH dehydrogenase, succinate dehydrogenase, and ubiquinol cytochrome c reductase (coenzyme Q) in complexes I, II, and III, respectively, donate electrons that lead to superoxide anions, the precursors of most reactive oxygen species [15]. The formation of ROS in the mitochondria is caused by these proteins, as shown by studies in which inhibitors were used to block the individual complexes of the mitochondrial respiratory chain. However, the relative importance of each complex may vary depending on the specific context and conditions [16,17,18,19].

The hypoxic increase in intracellular ROS concentration ([ROS]_i_) occurs mainly through mitochondria and NOX [20,21]. Numerous studies have reported that chronic or acute hypoxia increases the production of H_2_O_2_ and O_2_^−^ in PAECs [22,23,24,25,26,27]. However, the precise molecular mechanisms, the primary sites of ROS formation, and the functional role of ROS in PAECs under hypoxic conditions are not yet fully understood. In contrast to other cells, including PASMCs, mitochondria in ECs are not thought to be the primary source of ROS formation [28]. Instead, NOX is thought to be the primary catalyst for the formation of ROS in ECs [27,29,30], with endothelial nitric oxide synthase (eNOS), xanthine oxidases, and lipoxygenases serving as secondary catalysts [29]. Nevertheless, some studies have demonstrated a primary role of mitochondria in the production of ROS in endothelial cells [20,31,32]. Several studies conducted by us and others using PASMCs have shown that complexes I, II, and III are involved in hypoxia-induced mitochondrial ROS formation [16,18,33,34,35], but complex III, whose catalytic subunit, Rieske iron–sulfur protein (RISP), serves as an essential component [36,37,38].

Hypoxia-induced ROS have been associated with endothelial cell proliferation and migration [22,39,40,41], two essential processes involved in the physiopathology of PH. Moreover, ROS in endothelial cells are an important factor in many diseases, including cigarette smoke-induced chronic obstructive pulmonary disease (COPD) and pulmonary hypertension [42,43]. In addition, chronic hypoxia (CH) exacerbates the deleterious effects of cigarette smoke on the pulmonary circulation by increasing pulmonary arteriolar remodeling [44]. In this study, we investigated the nature of ROS signaling in human PAECs (HPAECs) chronically exposed to hypoxia. We also observed a significant increase in the proliferation of HPAECs under hypoxic conditions, which was inhibited by knocking down mitochondrial RISP, suggesting that mitochondrial ROS generation is involved in hypoxia-induced cell proliferation of PAECs. Interestingly, nicotine, a major component of cigarette smoke, was found to promote the proliferation of PAECs, probably through ROS-dependent mechanisms. Overall, our data indicate that hypoxia-induced production of ROS in human PAECs is regulated by RISP in mitochondria and that mitochondrial ROS may contribute to the functional role of endothelial cells in cigarette smoke-induced pulmonary hypertension. Understanding the molecular mechanisms underlying the production of ROS in PAECs and their functional role in hypoxia-PH could lead to the development of new therapeutic strategies for this devastating disease.

## 2. Materials and Methods

### 2.1. Cell Culture

Primary human PAECs were purchased from Lonza (Allendale, NJ, USA) and cultured in Lonza’s EGM-2 media with growth factor (CC-3162 and CC-3129) in a humidified atmosphere of 5% CO_2_ and 95% air at 37 °C. For experimental procedures, cells were trypsinized in passages up to 11 and seeded into a 96-well plate containing 25,000 cells/well.

### 2.2. Hypoxia Exposure

Cells were exposed to Hank’s buffered saline solution (HBSS) containing 10 mM HEPES aerated with a gas mixture of 20% O_2_, 5% CO_2_, and 75% N_2_ (normoxia) or 1% O_2_, 5% CO_2_, and 94% N_2_ (hypoxia). To prevent the entry of atmospheric oxygen, the hypoxic HBSS was tightly sealed.

### 2.3. Detection of ROS Production

ROS production in the cells was first evaluated using the Amplex UltraRed reagent (ThermoFisher/Molecular Probes, Waltham, MA, USA) to measure H_2_O_2_ in the medium. Amplex UltraRed reagent (50 μM) was added to the wells. After 20 min of incubation, the emitted fluorescence was measured using a FlexStation III microplate reader (Molecular Device, Sunnyvale, CA, USA) with 490 nm excitation and 540 nm emission.

To confirm the aforementioned dye-based assays, we further measured ROS production using the genetically encoded H_2_O_2_ sensor HyPer (Axxora (Farmingdale, NY, USA)). This specific biosensor consists of a regulatory domain of the transcription factor OxyR (OxyR-RD) inserted into a circularly permuted yellow fluorescent protein (YFP). OxyR-RD binds selectively to H_2_O_2_, undergoes a significant conformational change due to the formation of an intramolecular disulfide bond between two cysteine residues, and then shifts its excitation spectrum to allow HyPer to act as a specific H_2_O_2_ probe. In this assay, cells at 70–80% confluence were transfected with 0.8 μg pHyPer-dMito, a mammalian expression vector encoding mitochondria-targeted HyPer (HyPer-mito), 2 μL P3000 reagent, and 1 μL Lipofectamine 3000 for 2 days. ROS production in mitochondria was evaluated by taking HyPer-derived fluorescence images using an LSM510 laser scanning confocal microscope (Zeiss, New York, NY, USA). Images were taken with a 63× oil objective.

### 2.4. Knockdown of Rieske Iron–Sulfur Protein

We employed lentiviral short hairpin RNAs (shRNAs) targeted at RISP to knock down its expression in HPAECs, as described in our previous publications [38,45]. We used Thermo Scientific OpenBiosystems lentiviruses that contained RISP shRNAs and non-silence shRNAs, which were subsequently packaged using the pCMV-dR8.2 dvpr and pCMV-VSV-G packing vectors. Western blot experiments were performed to assess the effectiveness of the knockdown of RISP.

### 2.5. Scratch Wound Assays

Human PAECs were cultured to 80% confluence. After 48 h of exposure to hypoxia, the cells were carefully washed, and the culture media replaced. In another series of experiments, the cells were either exposed to 3 μM nicotine or exposed to hypoxia and nicotine simultaneously for 48 h. A linear wound was created by carefully and continuously aspirating cells using a sterile pipette tip. Images at 0, 12, and 18 h after the wound were taken with a 10× objective through an inverted microscope. Using the ImageJ2 program and MiToBo plugging, the wound area was measured in each image at the 18 h time point. Each measurement is the average of at least three different measurements. The data are presented as the ratio of the restored area and the initial time (0), which was determined by calculating the initial wound area minus the wound area at the specified time point and then dividing by the initial wound area.

### 2.6. Cell Proliferation Assays

In vitro assessment of HPAEC proliferation was done using Ki67 expression. HPAECs were stained in accordance with the manufacturer’s instructions using a labeled streptavidin biotin detection kit (Histo stain-plus kit; Zymed Laboratory Inc., South San Francisco, CA, USA). Using Ki67 nuclear staining, which produces a brown tint, a blinded pathologist identified the positive cellular state. By dividing the number of cell nuclei that express Ki67 by the total number of cell nuclei, the percentage of Ki67-positive cells was calculated.

We also assessed cell proliferation using direct cell count. The cell numbers were determined by directly counting the number of cells with a hemocytometer under a light microscope for 24 h and growth arrest with serum-free media for another 24 h. The formula for calculating the percentage of cell growth was (cell number in treatment group/cell number in control group).

### 2.7. Statistical Analysis

Data in this manuscript are expressed as mean ± the standard error of the mean (S.E.M.). Each experiment was conducted independently. For the same samples, a paired Student’s *t* test was used to assess significant differences before and after treatments. For different samples, an unpaired Student’s *t* test for two separate groups, and a one- or two-way ANOVA with the appropriate post hoc test for multiple comparisons were all performed to determine their statistical significance. Statistics were judged significant at *p* < 0.05.

## 3. Results

### 3.1. Inducing Hypoxic Conditions Can Result in a Large Increase in ROS Production in HPAECs

**Figure 1:**
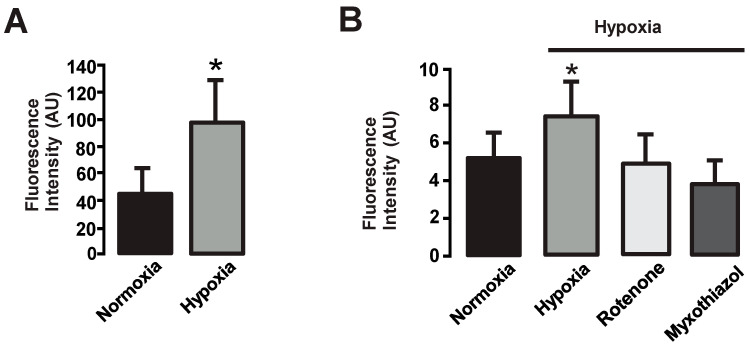
Hypoxia increases the formation of ROS in human pulmonary artery endothelial cells, and mitochondrial complex I and complex III are responsible for ROS production. (**A**) Exposure to hypoxia for 10 min significantly increased the formation of H_2_O_2_ in human PAECs. Cells were incubated with Amplex UltraRed (50 µM) for 20 min. The fluorescence produced by Amplex UltraRed was measured using the FlexStation III reader as an indicator of H_2_O_2_ production. Data are from three different experiments and are expressed as mean ± S.E.M. * *p* < 0.05 compared to normoxia, *n* = 4. (**B**) Cells were transfected with HyPer for 2 days, treated for 10 min without (control) and with rotenone (10 µM) or myxothiazol (10 μM), and then exposed to hypoxia. The bar graph illustrates that hypoxia enhanced the formation of H_2_O_2_ in human PAECs and rotenone and myxothiazol blocked this response, suggesting a role of complex I and complex III in this phenomenon. HyPer-derived fluorescence was measured using an LSM510 confocal microscope. Data are expressed as the media ± S.E.M. and were obtained from at least 50 cells in each group. * *p* < 0.05 compared to normoxia, *n* = 5.

We first investigated whether a hypoxic stimulus for 10 min could increase the production of ROS in HPAECs using different methods. We used Amplex UltraRed reagent to measure the formation of hydrogen peroxide (H_2_O_2_). The results showed that hypoxia exposure caused a significant increase (by ~50%) in Amplex UltraRed-derived fluorescence, indicating the production of H_2_O_2_ in human PAECs (Figure 1A).

### 3.2. Mitochondria Play an Important Role in ROS Generation in HPAECs After Hypoxia

Mitochondria are known to be one of the major sources of [ROS]_i_ in several cell types, including neurons [46], cardiomyocytes [47], hepatocytes [48], and pulmonary artery smooth muscle cells [38]. However, mitochondria were not considered important for ROS production in endothelial cells (ECs) [28,29]. Therefore, we sought to investigate the formation of ROS in the mitochondria of HPAECs after 10 min of hypoxia exposure. We focused on H_2_O_2_ rather than O_2_^−^ because H_2_O_2_ is known to be a stable and diffusible ROS molecule [49,50]. To illustrate the role of mitochondrial complexes in the production of ROS in HPAECs, cells were treated for 10 min without (control) and with rotenone (inhibitor of complex I, 10 µM) or myxothiazol (inhibitor of complex III, 10 mM), and then exposed to hypoxia. We have previously demonstrated in pulmonary artery smooth muscle cells (PASMCs) that mitochondrial complexes I, II, and III actively produce ROS in response to hypoxia, although complex III appears to be more important [35,36,45]. As shown in Figure 1B, rotenone and myxothiazol blocked the increase in ROS in HPAECs, suggesting that mitochondrial complexes I and III are involved in the formation of ROS induced by hypoxia in these endothelial cells, although complex III seems more important.

### 3.3. Hypoxia, Nicotine, and Hypoxia in Co-Exposure with Nicotine Increase the Mitochondrial ROS Concentration ([ROS]_m_) in HPAECs

**Figure 2:**
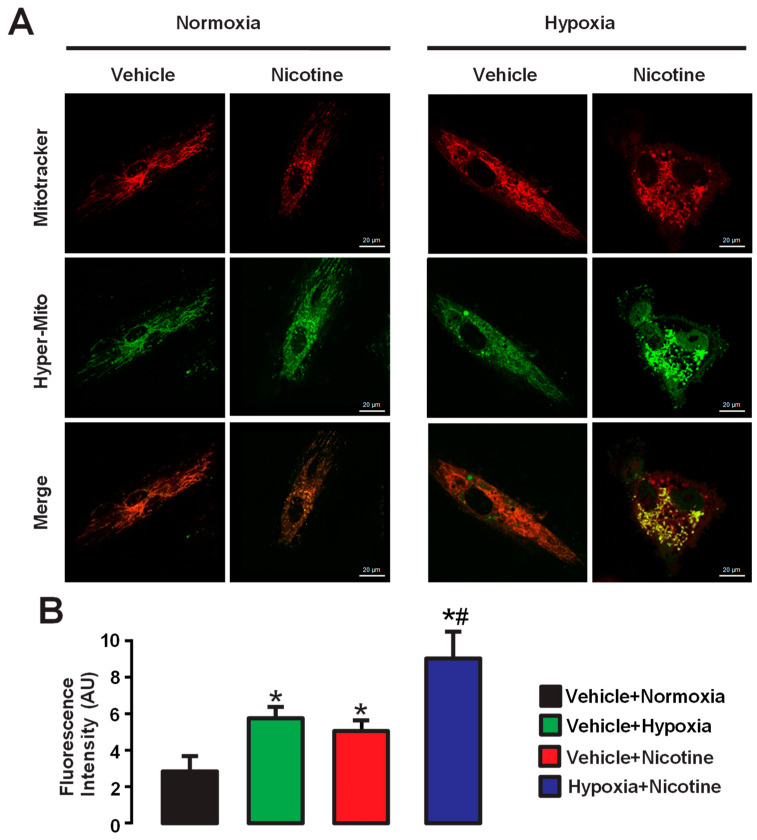
Hypoxia, nicotine, and hypoxia + nicotine for 2 days increase the formation of ROS in isolated mitochondria from human pulmonary artery endothelial cells. (**A**) HPAECs were treated with vehicle (control) or nicotine under a normoxic or hypoxic environment. MitoTracker was used to stain mitochondria and is shown in red color. The second row shows the detection of H_2_O_2_ and O_2_^−^ with HyPer and MitoSox, respectively, in green color. The third row shows composite images from the previous panels. The second row shows cells treated with 3 μM nicotine for two days under normoxic conditions, and the fourth column shows cells treated with hypoxia and nicotine. Note that when cells are exposed to hypoxia or nicotine, increased fluorescence is observed in the second row (HyPer-mito), i.e., increased ROS are produced. In addition, simultaneous exposure of the cells to hypoxia and nicotine led to even higher fluorescence values. The merged images show the colocalization of ROS production after hypoxia or nicotine and mitochondria. (**B**) The bar graph summarizes the changes in fluorescence intensity in arbitrary units (AU) of cells exposed to nicotine 3 μM, hypoxia, or nicotine + hypoxia for two days. Data are expressed as media ± S.E.M., *n* = 4. * *p* < 0.05 compared to control cells (vehicle + normoxia). ^#^ *p* < 0.05 compared with vehicle + hypoxia and nicotine + normoxia (vehicle) groups.

Complementary to the chemical dye-based hypoxia-induced ROS production in mitochondria, we further determine the effect of hypoxia on mitochondrial ROS production in HPAECs using genetically encoded H_2_O_2_ biosensor HyPer. In these experiments, cells were transfected with HyPer-mito to determine H_2_O_2_ generation in mitochondrial areas, and cells were also stained with MitoTracker to indicate mitochondrial areas (Figure 2A). Similar to the aforementioned results, hypoxia caused a significant increase in mitochondrial H_2_O_2_ generation in control cells. We have also found that nicotine, like hypoxia, produced a similar increase in mitochondrial H_2_O_2_ generation. Moreover, simultaneous treatment with nicotine and hypoxia caused a larger increase in mitochondrial ROS generation. Once we established the role of mitochondrial complexes I and III in the formation of ROS in HPAECs, we decided to evaluate the production of ROS in mitochondria by using MitoTracker. In addition, chronic nicotine use has been associated with changes in pulmonary and systemic blood pressure and right ventricular remodeling, which may lead to the development and progression of PH [51]. Nicotine may cause endothelial dysfunction and alter vasoreactivity through endothelium-dependent mechanisms, two factors that promote cardiovascular disease [52,53,54]. Moreover, ROS in endothelial cells is a major factor in many diseases, including cigarette smoke-induced chronic obstructive pulmonary disease (COPD) and PH [55,56]. Therefore, we investigated the effect of co-exposure of hypoxia and nicotine on the production of ROS in the mitochondria of HPAECs. To test the effect of hypoxia and nicotine, cells were first transfected with HyPer or MitoSox and Lipofectamine 3000 for 2 days and then stained with MitoTracker for 30 min. This procedure allowed us to assess the increase in _mt_ROS in HPAECs. As shown in Figure 2A,B, fluorescence intensity increased after hypoxia or nicotine exposure (3 µM, for two days), indicating that _mt_ROS production was augmented. The combination of hypoxia and nicotine for two days synergistically potentiated the increase in the production of ROS induced by either hypoxia or nicotine alone (Figure 2B).

### 3.4. Hypoxia Alone and in Co-Exposure with Nicotine Triggers the Migration of HPAECs

**Figure 3:**
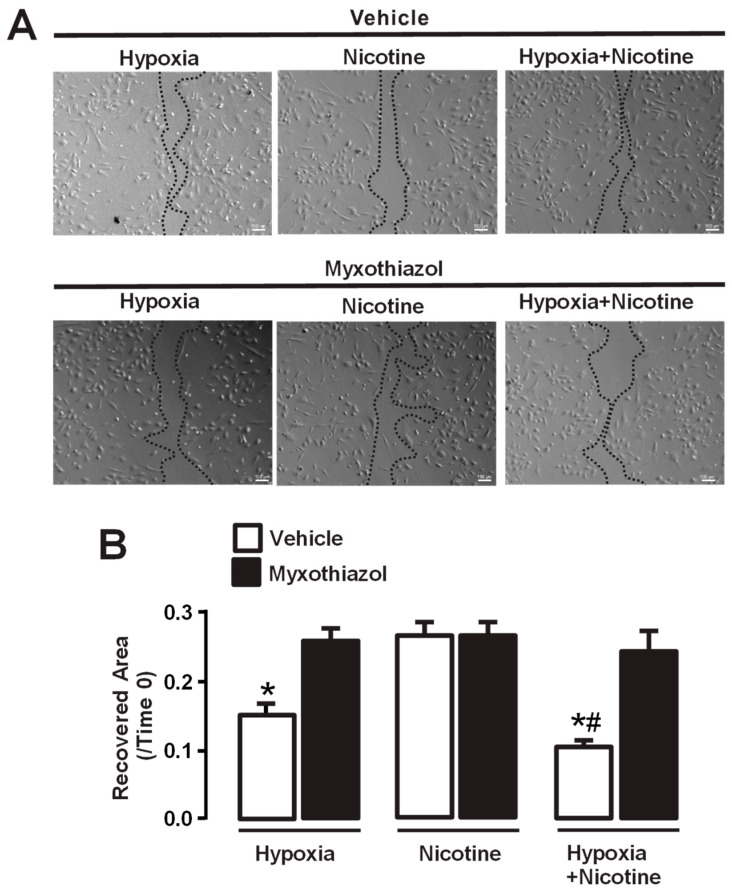
Effect of myxothiazol treatment on migration of human pulmonary endothelial cells (HPAECs). (**A**) Representative images of scratch wound experiments showing migration of pulmonary artery endothelial cells under different conditions. Hypoxia indicates cells exposed to hypoxic conditions for two days. Nicotine represents cells treated with nicotine (3 μM), and hypoxia + nicotine indicates cells treated with nicotine (3 μM) and exposed to hypoxic conditions. These groups of experiments were treated with myxothiazol or with the vehicle. Dashed lines depict the edge migration distance. (**B**) Quantification of the restored area in the scratch wound assay. The percentage of area recovered was calculated for each condition. The bar chart shows the percentage of recovered areas in the scratch wound test over a period of 18 h, depicted as a percentage of the area that remained open (ratio of recovered area/time). Hypoxia significantly increased cell migration compared to the control group, and the treatment with myxothiazol effectively blocked hypoxia-induced cell migration. Nicotine did not modify the migration of endothelial cells; however, the combination of hypoxia and nicotine further enhanced cell migration, which was also significantly inhibited by myxothiazol. Data are expressed as media ± S.E.M., *n* = 5. * *p* < 0.05 compared with controls (vehicle). ^#^ *p* < 0.05 compared between hypoxia + nicotine group vs. hypoxia.

The migration of PAECs plays a significant role in pulmonary vascular remodeling observed in PH [57]. Studies have demonstrated that exposure to hypoxic conditions can trigger the migration of vascular endothelial cells [58]. Using a scratch wound healing assay, we exposed HPAECs to a hypoxic stimulus for two days and examined migration at the 18 h time point. The cell migration was determined by the extent of the recovered wound area, which was calculated by dividing the scratch-evoked wound area at the 18 h time point by that at the initial time (0 h). Our data indicate that the hypoxic stimulus significantly induced endothelial cell migration (Figure 3A,B). In contrast, nicotine at a concentration of 3 µM did not elicit an increase in cell migration compared to hypoxia. These findings indicate that nicotine alone does not directly influence the migratory capacity of pulmonary endothelial cells. Moreover, we further examined the combined effects of hypoxia and nicotine on cell migration. The data indicate that the hypoxia and nicotine combination produced a similar effect to hypoxia alone, suggesting no synergistic pathway (Figure 3).

To explore the underlying mechanisms of hypoxia-induced cell migration, we treated the cells with myxothiazol, an inhibitor of the mitochondrial complex III. Inhibition of the mitochondrial complex III to block its derived ROS generation could effectively decrease hypoxia-induced endothelial cell migration; as such, in the presence of myxothiazol, a significantly reduced recovered wound area was observed (Figure 3A,B). These findings point out the crucial involvement of the mitochondrial complex III-derived ROS in the cell migration in response to hypoxic stimuli. In addition, the presence of myxothiazol neither significantly blocked the cell migration in the presence of nicotine nor produced a further effect in the co-exposure of hypoxia and nicotine.

### 3.5. Knockdown of RISP Reduces Hypoxia- and Hypoxia + Nicotine-Induced Migration of HPAECs

**Figure 4:**
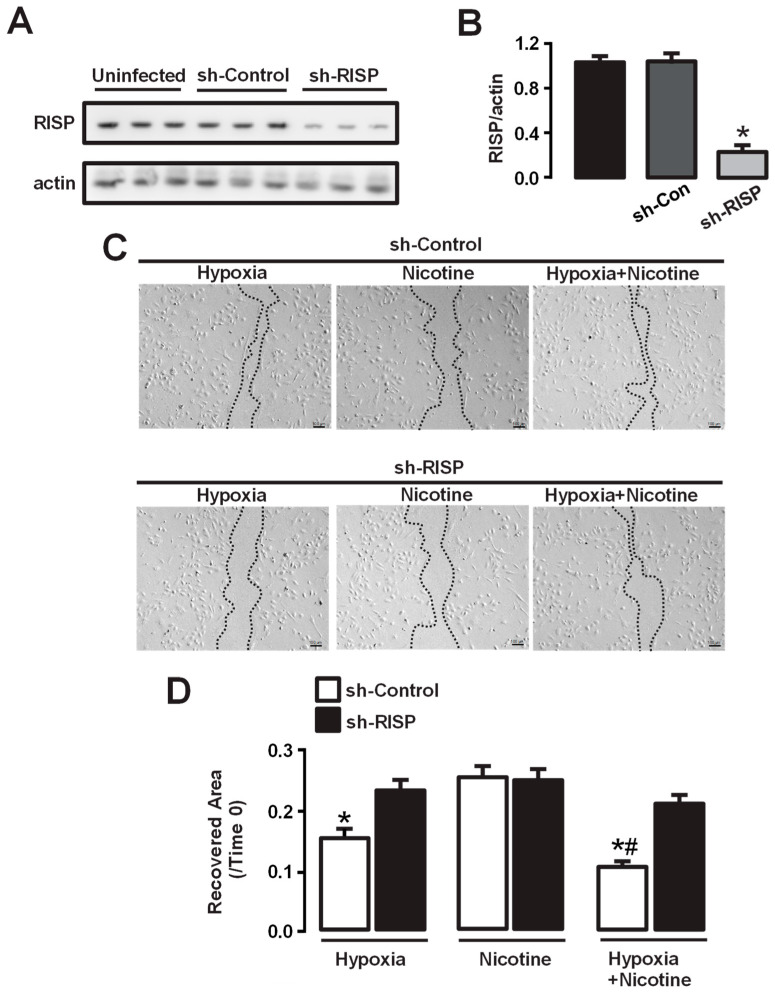
Rieske iron–sulfur protein (RISP) is involved in the hypoxia-induced migration of human pulmonary endothelial cells (HPAECs). (**A**) Representative blots illustrate the expression of RISP under different conditions: uninfected, infected with lentiviral non-silencing shRNAs (sh-Control), and infected with lentiviral shRNAs targeting RISP (sh-RISP). The second row depicts the expression of actin as a loading control. (**B**) The bar graph summarizes the densitometric analysis, showing that the expression of RISP was not altered when the cells were infected with non-silencing shRNAs (sh-Control) but was almost abrogated when treated with sh-RISP. The relative expression of RISP was quantified using the loading control (actin). (**C**) The images illustrate the migration of pulmonary artery endothelial cells after the indicated treatments. The control group showed minimal migration, while hypoxia stimulation and the combination of hypoxia and nicotine induced remarkable cell migration. RISP knockdown (sh-RISP) attenuated cell migration. Dashed lines depict the edge migration distance. (**D**) Quantification analysis shows the percentage of restored area in the scratch wound experiment. The graph illustrates the percentage of recovered areas in the scratch wound assay over a period of 18 h, depicted as a percentage of the area that remained open (ratio of recovered area/time). Hypoxia significantly increased cell migration compared to the control group. Nevertheless, the knockdown of RISP noticeably decreased the cell migration induced by hypoxia. Moreover, the combination of hypoxia and nicotine further increased cell migration, which was significantly inhibited by the knockdown of RISP. Data are expressed as media ± S.E.M. * *p* < 0.05 compared with control cells (vehicle or sh-Control). *n* = 4–5. ^#^ *p* < 0.05 compared between hypoxia + nicotine groups vs. hypoxia groups.

There is evidence of a link between Rieske iron–sulfur protein (RISP), ROS formation, and pulmonary hypertension [4,45,56,59]. RISP, a catalytic subunit of complex III, is known to be a primary molecule in mitochondrial ROS generation [4,36,37,45]. Therefore, we aimed to investigate the role of RISP in hypoxia- and nicotine-induced migration of endothelial cells. As described in our previous publications, infection with lentiviral shRNAs designed for RISP significantly decreased its protein expression in PASMCs [36,38,60]. Three different experiments with HPAECs showed comparable results. The downregulated expression of RISP was verified by Western blot (Figure 4A,B). In contrast, infection with lentiviral non-silencing (control) shRNAs did not lead to abrogation of RISP expression (Figure 4A,B).

We observed that exposure to hypoxic conditions for two days resulted in a significant increase in cell migration compared to the control group (Figure 4C,D). However, the knockdown of RISP caused a notable attenuation of cell migration (Figure 4D). These findings suggest that RISP is crucial for the migration response of pulmonary endothelial cells to hypoxic stimuli. In addition, we compared the effects of nicotine on cell migration in the absence of RISP. Similar to our previous observations, nicotine alone did not significantly improve cell migration (Figure 4C,D). However, the combination of hypoxia and nicotine still resulted in an increased migration response. Importantly, the enhanced migration triggered by the combination of hypoxia and nicotine was significantly blocked when RISP was genetically deleted, comparable to the effects observed with myxothiazol (Figure 4D).

### 3.6. Hypoxia Increases Cell Proliferation and Potentiates the Effect of Nicotine, a Major Component of Cigarettes and a Strong ROS Inducer

**Figure 5:**
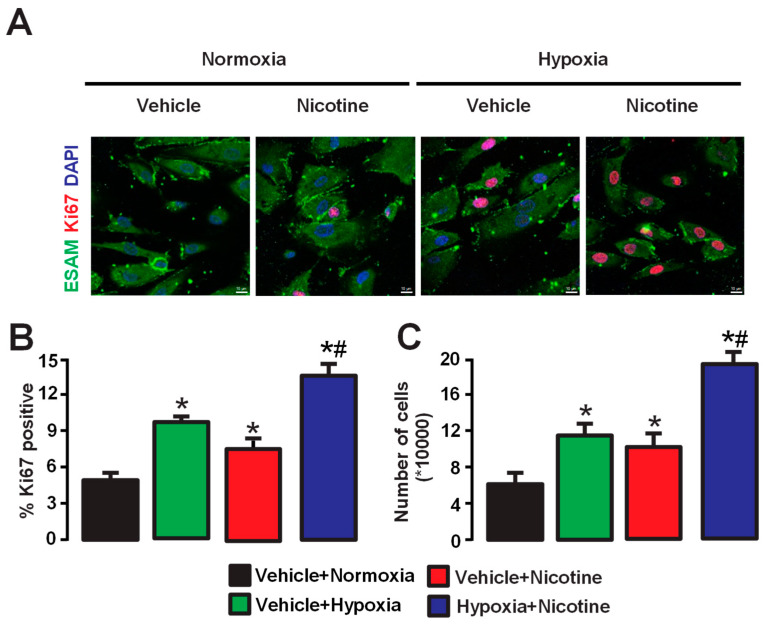
Hypoxia, nicotine, and hypoxia + nicotine for 2 days increase the proliferation of human pulmonary artery endothelial cells (HPAECs). (**A**) HPAECs were seeded in 24-well plates and reached 80% confluence. In the first column, cells were treated with the vehicle in a normoxic environment. In the second column, cells were treated with nicotine. All groups were stained with Ki67 to detect cell proliferation (shown in red). The cell nuclei were counterstained with DAPI and are shown in blue. Also, the endothelial cell-selective adhesion marker (ESAM) was used to confirm the identity of endothelial cells (green). Note that when cells were exposed to hypoxia, nicotine, or a combination of both, increased fluorescence of Ki67 was observed compared to the control group (vehicle + normoxia). (**B**) The bar graph quantifies the percentage of Ki67-positive cells in each sample: cells exposed to hypoxia, nicotine 3 μM, or hypoxia + nicotine for two days. Note that hypoxia or nicotine 3 μM increased the percentage of Ki67-positive cells, while a higher percentage of positive cells is observed when cells are exposed to hypoxia and nicotine. (**C**) The bar graph illustrates the changes in the number of cells exposed to hypoxia, nicotine 3 μM, or the combination of both treatments using a cell counting assay. The maximum number of cells was observed when cells were treated with hypoxia and nicotine. Note that hypoxia alone increased the cell number by ~100% (from 6 × 10^4^ to 12 × 10^4^), while the combination of hypoxia + nicotine increased the cell number from 6 × 10^4^ to 18 × 10^4^. Data are expressed as the media ± S.E.M. * *p* < 0.05 compared to control cells (vehicle + normoxia), *n* = 5. ^#^ *p* < 0.05 compared to the nicotine + normoxia and vehicle + hypoxia groups.

Both hypoxia and nicotine have been shown to promote the growth and proliferation of PAECs in vitro [22,61]. Endothelial cell proliferation plays an essential role in the development of PH [62]. Here, we performed a Ki67 proliferation assay and found that hypoxia increased the proliferation of HPAECs by approximately twofold, i.e., the cell number was increased from 6 × 10^4^ to 12 × 10^4^ (Figure 5A,B). Nicotine also increased the proliferation of HPAECs, and the simultaneous exposure of hypoxia and nicotine enhanced the proliferation of human PAECs threefold (Figure 5A,B). The results of the cell-counting assay, summarized in Figure 5C, showed that both hypoxia and nicotine independently increased the number of cells, and that hypoxia further enhanced the effect of nicotine on cell growth.

### 3.7. Pharmacological Inhibition of Mitochondrial Complex III Reduces Hypoxia- and Nicotine-Induced Proliferation of HPAECs

**Figure 6:**
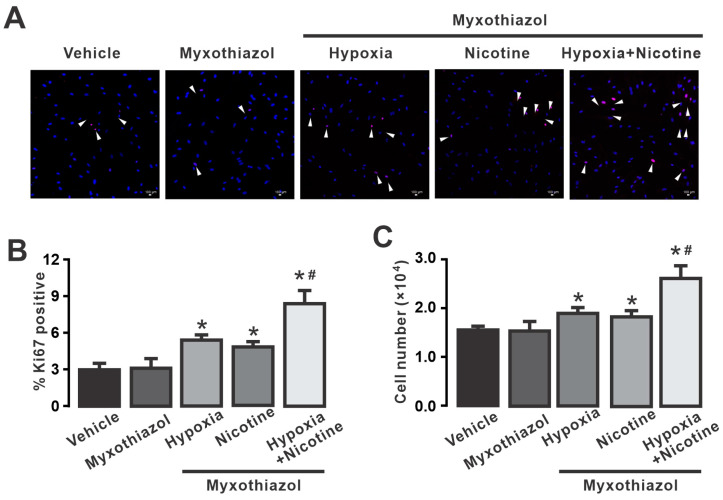
Inhibition of mitochondrial complex III reduces cell proliferation induced by hypoxia, nicotine, or nicotine plus hypoxia in human pulmonary artery endothelial cells (HPAECs). (**A**) Effect of myxothiazol on hypoxia- and nicotine-induced increased cell proliferation. In the first column, cells were treated with the vehicle. The second column illustrates the treatment with myxothiazol. In the last three columns, cells were exposed to hypoxia or nicotine (3 µM) or both (for two days) after the use of myxothiazol. Note that the effect caused by the combination of hypoxia and nicotine was not altered by myxothiazol. The white arrows indicate Ki67-positive endothelial cells (red). The cell nuclei were counterstained with DAPI (blue). (**B**) The bar graph summarizes the changes in the percentage of Ki67-positive endothelial cells treated with or without myxothiazol and exposed to hypoxia, nicotine 3 μM, or hypoxia + nicotine. Hypoxia and nicotine separately slightly increased the percentage of Ki67-positive cells in the presence of myxothiazol; however, co-exposure of hypoxia and nicotine still caused a significant augment of Ki67-positive cells. (**C**) The bar graph quantifies the total number of HPAECs incubated with myxothiazol and treated with hypoxia, 3 μM nicotine, or hypoxia + nicotine as determined by cell counting. Note that inhibition of mitochondrial complex III with myxothiazol significantly reduced the increase in cell number caused by hypoxia or nicotine. Data are expressed as media ± S.E.M. * *p* > 0.05 compared to control cells (control), *n* = 5. ^#^ *p* > 0.05 compared between hypoxia + nicotine groups and hypoxia or nicotine groups.

Mitochondrial complex III plays a pivotal role in the formation of ROS [63] and the proliferation of endothelial cells [64]. Our results showed that a two-day exposure to both hypoxia and nicotine at a concentration of 3 μM increased the percentage of Ki67-positive endothelial cells compared with the vehicle (Figure 5). Moreover, the combination of hypoxia and nicotine further enhanced this effect, suggesting a synergistic interaction. However, pretreatment with myxothiazol before exposure to hypoxia, nicotine, or their combination led to a reduced increase in cell proliferation (Figure 6) as compared with that shown in Figure 5. Figure 6B illustrates that when cells were pretreated with myxothiazol, hypoxia, or nicotine, this only caused a slight increase in Ki67-positive cells. Similarly, in the presence of this drug, hypoxia or nicotine only increased the cell number from 1.5 × 10^4^ to 1.9 and 1.8 × 10^4^, respectively (Figure 6C).

### 3.8. Genetic Downregulation of Rieske Iron–Sulfur Protein Largely Diminishes Hypoxia- and Nicotine-Induced Proliferation of HPAECs

**Figure 7:**
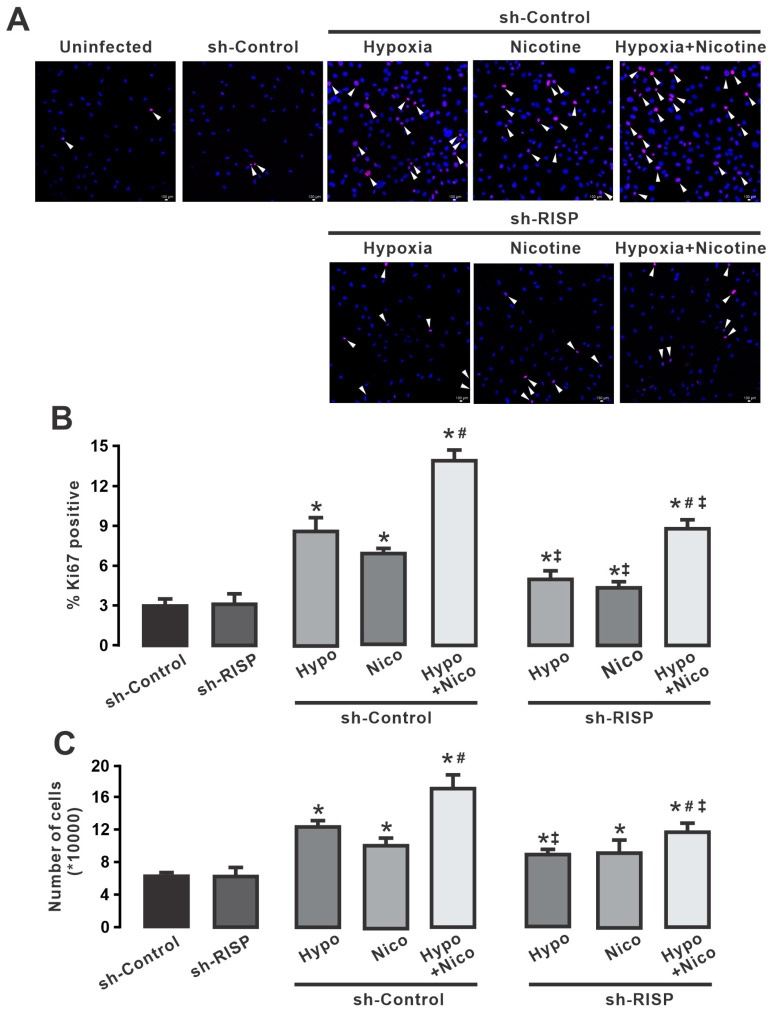
Knockdown of RISP significantly decreases hypoxia-, nicotine- and simultaneous exposure of hypoxia and nicotine-induced cell proliferation in human pulmonary artery endothelial cells (HPAECs). (**A**) Immunofluorescence studies showed a decrease in Ki67-positive endothelial cells exposed to hypoxia or nicotine and infected with lentiviral sh-RISP. In the first series, cells were uninfected, infected with sh-Control, and infected with sh-Control and exposed to hypoxia, nicotine 3 μM, or hypoxia + nicotine. Note that exposure to hypoxia or nicotine resulted in an increase in endothelial cell proliferation and the combination of hypoxia + nicotine enhanced this increase. The white arrows indicate Ki67-positive endothelial cells (red). The cell nuclei were counterstained with DAPI (blue). The second row shows endothelial cells infected with lentiviral RISP shRNAs and treated with hypoxia, nicotine 3 μM, or hypoxia + nicotine. Note that the knockdown of RISP decreased the number of Ki67-positive endothelial cells under the three different conditions, as shown by the white arrows. (**B**) Quantification of the proportion of Ki67-positive endothelial cells infected with sh-Control or sh-RISP and treated with or without hypoxia, nicotine 3 μM, or hypoxia + nicotine. Hypoxia or nicotine increased the percentage of Ki67-positive cells when infected with sh-Control, and hypoxia + nicotine enhanced this response. However, suppression of RISP (sh-RISP) significantly reduced the hyperproliferation induced by hypoxia, nicotine, or hypoxia + nicotine. (**C**) The bar graph summarizes the total number of cells determined by cell counting of HPAECs infected with sh-Control or sh-RISP and exposed to hypoxia, nicotine 3 μM, or hypoxia + nicotine. Note that infection with lentiviral RISP shRNAs significantly reduced the increase in cell number caused by exposure to hypoxia or hypoxia + nicotine. However, the knockdown of RISP failed to reduce the increased number of endothelial cells induced by nicotine. Data are expressed as media ± S.E.M. * *p* < 0.05 compared to control cells (sh-Control), *n* = 5. ^#^ *p* < 0.05 in comparison between hypoxia + nicotine (Hypo + Nico) groups and nicotine (Nico) or hypoxia (Hypo) groups. ^‡^ *p* < 0.05 comparing sh-RISP Nico, Hypo, and Hypo + Nico groups vs. sh-Control Nico, Hypo, and Hypo + Nico groups.

Next, we explored the role of RISP on hypoxia-induced proliferation of human pulmonary endothelial cells. To this end, we knocked down the expression of RISP (sh-RISP). Consistent with the abrogation of RISP expression in HPAECs, cell proliferation triggered by hypoxia and nicotine was greatly reduced with lentiviral RISP shRNAs, as shown by immunofluorescence studies (Figure 7A). As illustrated in Figure 7B, hypoxia, and nicotine (3 μM) independently, increased the percentage of Ki67-positive cells, and the combination of nicotine and hypoxia significantly enhanced this increase when the cells were infected with control RISP shRNAs (sh-Control). However, when HPAECs were infected with RISP shRNAs, the increase in the proportion of Ki67-positive cells exposed to nicotine or hypoxia was almost completely blocked. Moreover, infection with lentiviral RISP shRNAs largely reduced the effect caused by the combination of hypoxia and nicotine (Figure 7B). Similar results were observed when the cell number was calculated directly, i.e., the increase in endothelial cell number induced by hypoxia or the combination with nicotine was significantly reduced by the knockdown of RISP (Figure 7C). The use of RISP shRNAs failed to reduce the effect of nicotine on the number of HPAECs. Taken together, these results suggest that hypoxia-induced hyperproliferation of HPAECs depends on mitochondrial ROS generation and that the formation of ROS is mainly mediated by RISP.

## 4. Discussion

The aim of the present study was to investigate the role of ROS in hypoxia-induced proliferation and migration of pulmonary artery endothelial cells, which are crucial factors in the development of hypoxia-induced PH. Our studies show that two-day hypoxia exposure increased the formation of ROS (H_2_O_2_ and O_2_^−^) in the mitochondria of HPAECs. The increase in the formation of ROS was greatly reduced by the inhibitor of mitochondrial complex III, myxothiazol, indicating an essential role of this complex in the production of ROS. We also found that hypoxia promoted hyperproliferation and migration of HPAECs mainly via the formation of ROS in complex III. Pharmacologic inhibition or genetic deletion of RISP in mitochondrial complex III significantly decreased the proliferation and migration of endothelial cells induced by the hypoxic stimulus. In addition, we found that nicotine, a major component of cigarette smoke, favored the proliferation of HPAECs and enhanced hypoxia-induced hyperproliferation and migration, possibly through ROS-dependent mechanisms.

Hypoxia is an important factor in the development and progression of PH. It triggers a cascade of events leading to vascular remodeling, endothelial dysfunction, and abnormal proliferation of PAECs. Our study emphasizes the central role of ROS in mediating these pathological processes. ROS—the superoxide anion (O_2_^−^) and hydrogen peroxide (H_2_O_2_)—are highly reactive molecules involved in both physiological and pathological cellular processes. In the context of PH, increased ROS production has been observed in PAECs, leading to oxidative stress and subsequent endothelial dysfunction [65]. Our results are consistent with previous studies showing that hypoxia increases ROS production in various cell types, including PAECs [22,23,24,25,26,27]. We found that a 10 min hypoxic stimulus increased the production of H_2_O_2_ (Figure 1A) in human PAECs. In these experiments, we focused on measuring H_2_O_2_ rather than O_2_^−^ because H_2_O_2_ is relatively more stable compared to O_2_^−^ and has a longer half-life. The superoxide anion is very reactive and tends to convert rapidly into other reactive oxygen species, such as H_2_O_2_ [8,49,50]. By measuring H_2_O_2_, we could determine the cumulative effects of the formation of the superoxide anion and its subsequent reactions. An increased production of ROS in response to hypoxia is thought to contribute to pulmonary vasoconstriction and the development of PH [65,66]. It is known that ROS are produced by two major endogenous sources, namely NOX and the electron transport chain (ETC) in mitochondria (8). However, in endothelial cells, mitochondria are not considered the primary site for the generation of ROS, which may be due to the low content of mitochondria in this type of cell compared to other cells with higher energy requirements [67]. In this regard, it has been reported that in several mammals, the volume of mitochondria is significantly lower in endothelial cells compared to cardiomyocytes [68]. In different vascular beds, the mitochondrial content of endothelial cells may vary depending on their function. For example, endothelial cells at the blood–brain barrier have a higher mitochondrial content than non-neuronal tissues, such as endothelial cells from the heart, lung, renal glomerulus, and skeletal muscle [69]. Therefore, we investigated the role of mitochondria in the production of ROS and its consequences in HPAECs. In addition, we have investigated the role of mitochondrial complexes in the formation of ROS. In general, there are two key molecular mechanisms that have been proposed to increase mitochondrial ROS after hypoxia (electron leakage from the ETC and complex III dysfunction). During hypoxia, the electron flow through the ETC is disrupted, leading to an accumulation of electrons. These accumulated electrons can react directly with O_2_, leading to the formation of superoxide radicals in the mitochondria. In addition, the disturbed electron flow within complex III can cause an imbalance in electron transfer, leading to the formation of superoxide radicals [2]. However, the involvement of the different complexes in the formation of ROS in PAECs is not yet fully understood. It has been described that ROS production in bovine aortic endothelial cell mitochondria mainly depends on reverse transport to complex I and occurs through the Q-cycle in complex III [70]. In addition, complex III has been reported to be the major site of ROS formation after hypoxia in human umbilical vein endothelial cells (HUVECs) [71]. Complex II is involved in lysophosphatidylcholine-dependent production of ROS [72]. Previous reports from our group have shown that although complexes I, II, and III cause the formation of ROS under hypoxic conditions in PASMCs, complex III appears to be more important [35,36,38,45]. In this study, we found that inhibition of complex I with rotenone decreased the formation of H_2_O_2_ in HPAECs, and inhibition of complex III with myxothiazol decreased the formation of H_2_O_2_ to a greater extent (Figure 1B), pointing out a major role of complex III in ROS formation.

Since inhibitors of mitochondrial complexes reduced the formation of H_2_O_2_ in response to acute hypoxia in HPAECs, we decided to explore the formation of ROS in the mitochondria of these cells after a two-day exposure to hypoxia using MitoTracker. Chronic hypoxia due to various underlying conditions, such as COPD, interstitial lung disease, sleep apnea, or living at high altitudes, can lead to decreased oxygen levels in the blood over prolonged periods of time, which can contribute to the development of PH over time [22]. We observed that HPAECs exposed to hypoxia for two days increased mitochondrial production of H_2_O_2_ and O_2_^−^ (Figure 2A,B). However, Yang and Block [8] reported in porcine PAECs that hypoxia decreased intracellular H_2_O_2_ production and extracellular release of H_2_O_2_ and O_2_^−^ when endothelial cells were exposed to 0% O_2_ for 2 to 16 h [8]. In addition, Zulueta and colleagues [73] observed that exposure of bovine PAECs to 3% and 0% O_2_ resulted in a reduction in H_2_O_2_ release (29.6 ±1.3% and 4.2 ± 0.7%, respectively) compared with ECs exposed to normoxia (20% O_2_). Although neither study clarifies the molecular mechanism involved in reducing the formation and release of ROS, they postulate a possible role of oxidation or autoxidation systems [8,73]. The discrepancy between the results of the two studies and our results may be due to differences in the time the endothelial cells were exposed to hypoxia, the hypoxic conditions, and the sensitivity of the methods used to measure ROS formation. For example, Yang and Block measured the release of H_2_O_2_ after exposure to normoxia (room air and 5% CO_2_) or hypoxia (0% O_2_, 95% N_2_, 5% CO_2_) from 2 to 16 h [8], whereas we used 20% O_2_, 5% CO_2_, and 75% N_2_ for normoxic conditions or 1% O_2_, 5% CO_2_, and 94% N_2_ for hypoxia stimulus for 10 min or 2 days. In this context, the conditions used by Yang and Block refer to an anoxic state rather than hypoxia, which, together with the exposure time, could lead to the differences observed in our study. Similar oxygen deprivation conditions were used by Zuleta and colleagues, where a reduction in the production of ROS was observed when cells were exposed to anoxic conditions compared to hypoxia [73].

Chronic nicotine use, often associated with cigarette smoking, has been linked to the development and progression of PH [51]. For example, nicotine has vasoactive properties and can lead to pulmonary artery constriction, which promotes increased pulmonary vascular resistance, increased pulmonary artery blood pressure, and right ventricular remodeling [51,53,54]. Nicotine may impair the production and release of endothelium-derived relaxing factors such as nitric oxide and prostacyclin, so that endothelial dysfunction contributes to increased vasoconstriction and inflammation [53,74,75]. In addition, nicotine induces oxidative stress by promoting the production of ROS [54,76]. ROS may lead to endothelial dysfunction, smooth muscle cell proliferation, and pulmonary vascular remodeling, thus contributing to the progression of PH. In this study, we also found that the administration of nicotine 3 µM (for 2 days), a major component of cigarette smoke and agonist of nicotinic acetylcholine receptors (nAChRs), increased the formation of ROS in PAECs (Figure 2A,B). Moreover, the combination of hypoxia and nicotine further boosted the mitochondrial production of ROS (Figure 2B). The exact molecular mechanism by which nicotine increases mitochondrial ROS production is still an active area of research, and interconnected phosphorylation pathways might be involved. In this sense, it has been postulated that prolonged nicotine exposure elicits the opening of the mitochondrial transition pore, leading to enhanced ROS formation [77]. Also, chronic nicotine induces the phosphorylation of p66shc and its subsequent binding to cytochrome c in mitochondria, resulting in the production of H_2_O_2_ [78]. Furthermore, nicotine stimulation of Dihydro-β-erythroidine-sensitive nAChRs and activation of Akt and mitogen-activated protein kinase (MAPK) pathways could lead to the generation of mitochondrial ROS, which in turn stabilize and activate hypoxia-inducible factor (HIF)-1α [79]. Stabilized HIF-1α can activate genes that promote cell survival and proliferation, including those involved in angiogenesis and cell cycle progression. One of these mechanisms could be involved in the enhanced ROS generation elicited by the exposure of PAECs to hypoxia + nicotine observed in Figure 2B, although further research is needed.

Pulmonary hypertension is characterized by vascular remodeling of pulmonary arterioles caused primarily by the migration and proliferation of smooth muscle cells, fibroblasts, and endothelial cells [2,3]. Migration of PAECs is critical for angiogenesis, vascular remodeling, endothelial dysfunction, and the inflammatory response [6,80]. PAECs play a key role in arterial wall thickening due to excessive smooth muscle cell proliferation and vascular lumen narrowing by migrating from the inner lining of the pulmonary artery into the surrounding tissue [2,6]. Migration assays in this work showed that hypoxia triggered the migration of HPAECs, and the simultaneous administration of hypoxia and nicotine further increased the migration of HPAECs (Figure 3). We also found that myxothiazol and RISP knockdown significantly diminished migration induced by hypoxia or by the co-exposure of hypoxia and nicotine (Figure 3 and Figure 4). These findings provide further evidence for the crucial role of the mitochondrial complex III and, in particular, its subunit RISP in regulating the migratory capacity of lung endothelial cells. In this regard, it is well known that hypoxia and activation of HIF-1α cause the release of angiogenic signals (VEGF, ANG2, and FGF) from endothelial cells [81]. However, the precise molecular mechanism linking VEGF and ROS to induce endothelial cell migration has not been fully elucidated. For instance, in HUVECs, VEGF promotes ROS mitochondrial production and migration, a process involving the activity of the small GTPase Rac1 [41]. This protein is critical for the formation of lamellipodial structures and focal adhesion in response to VEGF, as well as for the attachment of cells to matrix proteins and the formation of tubular structures, explaining the role of Rac1 in endothelial cell migration [82].

Deregulated and prominent endothelial cell proliferation has been reported in plexiform lesions of pulmonary hypertension [62,83,84,85]. In addition, a marked proliferation of endothelial cells in pulmonary arterioles was observed in an animal model of pulmonary hypertension induced by hypoxia in combination with SU5416, an inhibitor of vascular endothelial growth factor (VEGF) receptors 1 and 2 [86]. Nevertheless, there is no convincing evidence that the proliferation of PAECs occurs in an animal model of hypoxia-induced pulmonary hypertension. Although some authors have reported no or adverse effects of hypoxia on PAEC proliferation [87,88], hypoxia-induced hyperproliferation has been demonstrated in human pulmonary microvascular endothelial cells in response to epidermal growth factor activation [89]. Moreover, PAECs from patients with idiopathic pulmonary arterial hypertension displayed augmented cell numbers in response to growth factors in culture compared with cells isolated from healthy donors [90]. In this work, we examined the effects of hypoxia and nicotine exposure on the proliferation and migration of HPAECs. After two days of hypoxia or nicotine treatment, the percentage of Ki67-positive cells was significantly increased (Figure 5), and direct cell counting showed similar results (Figure 5C). Importantly, the combination of hypoxia and nicotine further increased endothelial cell proliferation (Figure 5B,C), suggesting a synergistic mechanism.

Complex III is recognized to be an important factor in the formation of ROS in mitochondria. Although its contribution to the overall production of ROS may be relatively small compared with complex I, the electron leakage from complex III may directly interact with O_2_ to generate superoxide anions. Moreover, a blockade of complex III in lung endothelial cells impairs proliferation by decreasing the NAD^+^/NADH ratio [64]. Here, we report that pharmacological inhibition of the mitochondrial complex III with myxothiazol decreased proliferation induced by hypoxia or nicotine (Figure 6). RISP, one of the catalytic subunits of complex III, has been associated with alterations in mitochondrial function and increased ROS production and promotes the development and progression of pulmonary hypertension [4,38,56,59]. These changes may affect the smooth muscle cells and endothelial cells lining the pulmonary arteries, leading to vascular remodeling, vasoconstriction, and abnormal cell proliferation. Because hypoxia or nicotine enhanced endothelial cell proliferation in a manner dependent on the formation of ROS, we investigated the role of RISP in this phenomenon. Our results in Figure 7 shows that the knockdown of RISP strongly decreased endothelial cell proliferation induced by hypoxia, nicotine, or the combination of both treatments. In this context, chronic hypoxia (72 h) induces the formation of H_2_O_2_ and subsequently increases the expression of arachidonate 5-lipoxygenase (ALOX5) [22]. Pulmonary hypertension has been associated with 5-lipoxygenase and its downstream leukotriene derivatives [22,91]. The increase in ROS promotes the activity of p38 MAPK [92] and NF-κB [93], which in turn favor the activation and expression of ALOX5 [22]. Although the exact mechanism causing the increase in endothelial cell proliferation triggered by ALOX5 is not known, it is likely that this protein stimulates cell growth through its nuclear localization, interactions with cytoskeletal proteins, or VEGF signaling [22,94,95,96]. Moreover, STAT3 has been shown to be phosphorylated in endothelial cells from lesions of idiopathic pulmonary arterial hypertension, suggesting that activation of STAT3 contributes to the proliferative pulmonary vascular lesions in IPAH lungs [90], and it is known that ROS can activate STAT3, so it is likely that phosphorylated processes are involved in the ROS-mediated hyperproliferation of HPAECs. On the other hand, it has been suggested that the proliferative effects of nicotine are dependent on the activation of α7- and 9-α-nAChRs [97]. These receptors mediate the activation of PI3/Akt and Erk signaling pathways that promote cell growth [97,98]. As described earlier, these signaling cascades favor the production of ROS in mitochondria and the stabilization of HIF-1α [79]. Stabilized HIF-1α can activate genes that promote cell survival and proliferation, including those involved in angiogenesis and cell cycle progression. Regarding metabolic changes and hypoxia and ROS during PH, it is well known that anti-apoptotic phenotypes of endothelial cells, hyperproliferation, and cellular glycolytic reprogramming occur during PH [62,99]. Glycolytic reprogramming (a shift from oxidative phosphorylation to glycolysis) supplies the energy required for cell division and proliferation [100]. In addition, the glycolytic intermediates help to synthesize amino acids, phospholipids, and nucleotides needed for cellular reproduction. Due to this glycolytic reprogramming, the vascular cells in the pulmonary artery (including endothelial cells) proliferate abnormally [101,102]. One of the amino acids implicated in excessive cell proliferation is glutamine, and one of the most notable metabolic changes in vascular cells in response to hypoxia is glutamine and glutamate metabolism [103]. The impaired metabolism of glutamine may contribute to the pathogenic alteration of PH via redox homeostasis [3,104]. Dysregulated glutamine metabolism as the loss of glutathione may lead to an accumulation of ROS [105,106]. Interestingly, PH patients display increased glutamine uptake in the vasculature compared with control subjects [107]. Furthermore, after hypoxia, the stabilization of HIF-1α prevents glutamine from being oxidized [108], and HIF-2α can promote abnormal glutamine metabolism by triggering the PI3K/mTORC2 [109], which might lead to vascular cell proliferation. Thus, it is probablee that metabolic changes and increased ROS production after hypoxia could lead to the enhanced endothelial cell growth observed in this work.

## 5. Conclusions

Overall, the proliferation and migration of pulmonary artery endothelial cells is a complex process that contributes to vascular remodeling, endothelial dysfunction, angiogenesis, neovascularization, and inflammation in pulmonary hypertension. Interfering with the mechanisms involved in PAEC migration may have therapeutic potential for the treatment of this disease. Our results demonstrate the essential role of the mitochondrial complex III, particularly the Rieske iron–sulfur protein, in regulating hypoxia-mediated proliferation and migration of pulmonary endothelial cells. Inhibition of the mitochondrial complex III by myxothiazol and genetic suppression of RISP attenuate hypoxia- and nicotine-triggered cell proliferation and hypoxia-induced migration, highlighting their potential as therapeutic targets for modulating aberrant cell proliferation and migration in pulmonary vascular remodeling and pulmonary hypertension. In addition, our results suggest a potential synergistic interaction between nicotine and hypoxia in the induction of hyperproliferation and hypermigration of endothelial cells, further highlighting the complex interplay between these factors in pulmonary vascular remodeling and pathogenesis. Finally, our results suggest that RISP is a potential therapeutic target for interventions aimed at reducing abnormal cell proliferation and migration associated with pulmonary vascular remodeling and pulmonary hypertension.

## Data Availability

Data are available upon request.
